# Multiple Symmetric Lipomatosis (Madelung Disease) in a Large Canadian Family With the Mitochondrial *MTTK* c.8344A>G Variant

**DOI:** 10.1177/2324709618802867

**Published:** 2018-09-29

**Authors:** Uththara Perera, Brooke A. Kennedy, Robert A. Hegele

**Affiliations:** 1 Western University, London, Ontario, Canada

**Keywords:** adipose tissue, mitochondria, DNA sequencing, lipomatosis, lipodystrophy, monogenic

## Abstract

*Background.* Multiple symmetric lipomatosis (MSL), also known as Madelung disease, is a rare adult-onset disorder characterized by benign lipomatosis usually localized to the nuchal and upper thoracic region. A subset of these patients has germline variants in mitochondrial DNA. *Methods.* Three siblings of Northern European descent with MSL were assessed initially and provided whole blood for DNA analysis. Family history revealed several additional affected siblings who were dispersed across Canada. Targeted histories were obtained from 6 additional affected family members by telephone interviews using a standardized questionnaire, and genomic DNA was obtained from saliva. Sequencing of mitochondrial DNA was performed. *Genetic analysis.* Eight affected individuals who were studied each had the *MTTK* gene c.8344A>G variant. None of the affected individuals had epilepsy, ataxia, or myopathy. *Conclusion.* In this extended Canadian family, the rare *MTTK* c.8344A>G variant was linked with Madelung disease in multiple family members. Knowing the likely basis of MSL in this family may help with diagnosis, genetic counseling, monitoring for associated phenotypes, and potential future targeted interventions.

## Introduction

Madelung disease, or multiple symmetric lipomatosis (MSL; OMIM 151800), is a rare disorder of adipocyte differentiation that is characterized by benign, diffuse lipomatosis affecting cephalic, cervical, and upper thoracic subcutaneous depots.^1-5^ Although MSL has been primarily regarded as a cosmetic deformity, associated features sometimes include sensory, motor and autonomic neuropathy, and myopathy.^1-5^ Dyslipidemia, insulin resistance, hypothyroidism, hypertension, and renal and hepatic disease are relatively uncommon.^1^ Chronic excessive alcohol use has been linked with accelerated progression of lipomatosis in some MSL patients.^1^ A minority of patients have been reported to have large-scale deletions and specific point mutations within mitochondrial DNA (mtDNA), as well as in some nuclear genes encoding mitochondrial proteins, such as *MFN2* encoding mitofusin-2.^6,7^ In a multigenerational Canadian kindred with at least 8 family members with MSL, we report the complete co-segregation of the ultrarare mitochondrial gene *MTTK* c.8344A>G variant and the phenotype.

## Subjects and Methods

The proband and 2 siblings (subjects II-5, II-6, and II-7) were seen together in an endocrinology outpatient clinic regarding their lipomatosis. Medical histories were taken, and physical examinations were performed. Blood was collected for clinical biochemical studies including fasting glucose, lipid profile, insulin and C-peptide, thyroid, renal and liver function, hematology, coagulation, and for DNA extraction, as described.^8^

Six other living family members were identified as expressing MSL; due to geographical constraints, these individuals could not be directly evaluated in the clinic. Instead, telephone interviews were conducted for the remaining 6 subjects, including a targeted medical history via a standardized questionnaire (see Supplementary file; available in the online version of the article). DNA was obtained from 5 of these 6 affected family members: from blood in 1 subject (II-4) and from saliva samples from 4 subjects (II-2, II-3, III-3, and III-9), the latter were obtained using the Oragene DNA kit (OG-500; DNA Genotek, Ottawa, Ontario, Canada). The Western University Institutional Review Board approved the project (Reference #07290E), and the participants provided signed informed consent.

DNA was isolated from the whole blood or saliva from a total of 8 available family members. Samples from the 3 probands were analyzed for known genes in metabolism and lipodystrophy using the LipidSeq targeted next-generation sequencing panel.^9,10^ Primers for the *MTTK* gene were designed and used for both DNA amplification and Sanger sequencing (primer sequences and conditions available on request). Samples were amplified by polymerase chain reaction, and products were electrophoresed on a 1.5% agarose gel. The purified fragments were sequenced using the chain termination Sanger sequencing method (ABI 3730 Automated DNA Sequencer, Thermo Fisher Scientific, Ottawa, Ontario, Canada) at the London Regional Genomics Centre (www.lrgc.ca) using standard operating procedures and were analyzed using Sequence Navigator Software (PE-Applied Biosystems, Mississauga, Ontario, Canada).

## Results

### Case Summaries

**Figure 1.**
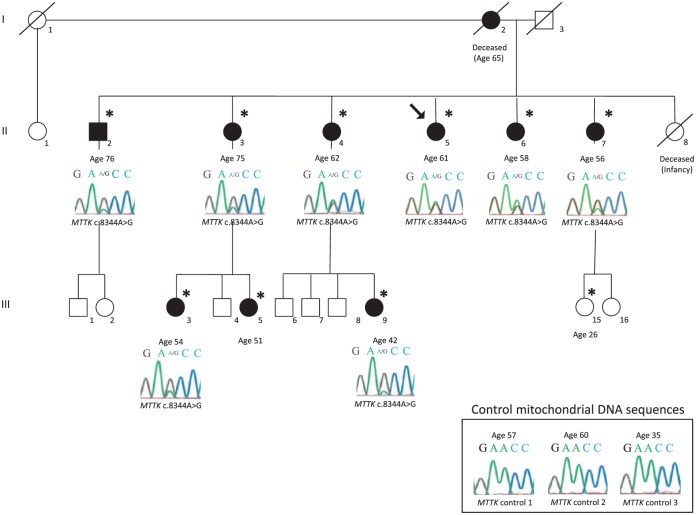
Pedigree structure of Canadian multiple symmetrical lipomatosis family. Pedigree showing the proband (II-5, arrow) in relation to 3 generations of family members. Males and females are squares and circles, respectively. Black shading indicates clinical affected status for multiple symmetric lipomatosis. Diagonal lines indicate deceased individuals. Asterisks indicate individuals who provided medical information. Ages of subjects who consented to participate are shown. Sanger sequence electropherogram tracings are shown for individuals who provided DNA samples for genotyping: the *MTTK* gene forward strand at nucleotide positions c.8342-8346 below each individual who provided a DNA sample. The guanine peak at position c.8344 confirms the presence of the *MTTK* c.8344A>G mutation in each sample. The presence of both guanosine and adenosine peaks confirms mitochondrial heteroplasmy, indicating more than one type of mitochondria in these cells. There is no relationship between the relative peak height and the severity of the clinical presentation (data not shown). Corresponding mitochondrial DNA sequences from 3 healthy control individuals, with ages indicated, are shown in the bottom right corner.

**Figure 2.**
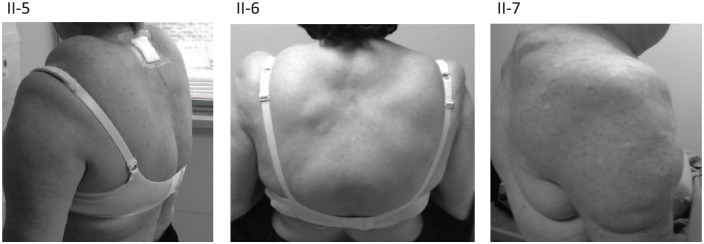
Affected regions of selected family members. Panels show the affected regions of subjects II-5, II-6, and II-7. There are both a few apparent discrete lipomas together with other areas of contiguous increased fat mass. The history in all affected family members is the same; initially there is appearance of discrete lumps, or recurrence of discrete lumps after surgical resection, liposuction, or other cosmetic procedure. With the passage of time, these can evolve in contiguous regions of increased fat mass.

Figure 1 shows the pedigree structure, including MSL status and electropherogram results. The family is of Anglo-Saxon ancestry. Figure 2 shows affected regions from subjects II-5, II-6, and II-7. Tables 1 and 2 summarize clinical and metabolic features of study participants.

**Table 1.** Summary of Clinical Features in Canadian Multiple Symmetrical Lipomatosis Family.

Individual Number	Age (Years)	Sex	*MTTK* c.8344A>G Variant	Cervical Lipomatosis	Generalized Seizures	Absence Seizures	Numbness/Tingling in Hands or Feet	Proximal Muscle Weakness	Balance Issues	Ataxia	Spasticity	Myopathy	Pes Cavus	Migraines	Dementia	Memory Loss	Ophthalmoparesis or Vision Loss	Hearing Loss^a^
I-2	D	Female	#	+	+	−	−	−	−	−	−	−	−	−	+	+	−	−
II-2	76	Male	+	+	−	−	+	−	+	−	−	−	−	−	−	−	−	−
II-3	75	Female	+	+	−	−	−	−	−	−	−	−	−	−	−	−	−	−
II-4	62	Female	+	+	−	−	+	−	−	−	−	−	−	+	−	+	+	−
II-5	61	Female	+	+	−	−	+	−	−	−	−	−	−	−	−	−	−	−
II-6	58	Female	+	+	−	−	−	−	−	−	−	−	−	−	−	−	−	−
II-7	56	Female	+	+	−	−	+	−	+	−	−	+	−	−	−	+	−	−
II-8	D	Female	#	−	−	−	−	−	−	−	−	−	−	−	−	−	−	−
III-3	54	Female	+	+	−	−	+	−	−	−	−	−	+	+	−	+	−	−
III-5	51	Female	#	+	−	−	+	−	−	−	−	−	−	−	−	−	−	−
III-9	42	Female	+	+	−	−	+	−	−	−	−	−	−	+	−	−	−	−
III-15	26	Female	#	−	−	−	−	−	−	−	−	−	−	−	−	−	+	−

Abbreviations: #, not tested, genotype unconfirmed; D, deceased.

a Hearing loss as reported by clinical history, audiometry not completed.

**Table 2.** Summary of Metabolic Features in Canadian Multiple Symmetrical Lipomatosis Family.

Individual Number	Age (Years)	Sex	*MTTK* c.8344A>G Variant	Cervical Lipomatosis	Diabetes Mellitus/Impaired Fasting Glucose	Dyslipidemia	Waist Circumference >94 cm (Male), or >80 cm (Female)	Hypertension	History of Excessive Alcohol Intake	Gout
I-2	D	Female	#	+	−	−	−	−	−	−
II-2	76	Male	+	+	+	−	−	+	−	+
II-3	75	Female	+	+	−	−	−	+	−	−
II-4	62	Female	+	+	−	−	−	+	−	−
II-5	61	Female	+	+	+	−	−	+	−	−
II-6	58	Female	+	+	−	+	−	+	−	−
II-7	56	Female	+	+	−	−	−	−	−	−
II-8	D	Female	#	−	−	−	−	−	−	−
III-3	54	Female	+	+	−	−	−	+	−	−
III-5	51	Female	#	+	−	−	−	−	−	−
III-9	42	Female	+	+	−	−	−	−	−	−
III-15	26	Female	#	−	−	−	−	−	−	−

Abbreviations: #, not tested, genotype unconfirmed; D, deceased.

Patient I-2 was the mother of 6 siblings who were studied. Her medical history included multiple symmetric lipomatosis, with age of onset at approximately 45 years, myoclonic epilepsy, schizophrenia, and dementia. She died of cancer at age 65.

Patient II-2 (current age 76) has the mildest clinical presentation of MSL, with small cervical lipomatous tumors whose onset was noted at approximately 60 years of age. The majority of the lipomatoses were successfully treated with dermolipectomy and liposuction at the time of diagnosis and have not grown or reoccurred since. Other medical history included a myocardial infarction at age 60, followed by coronary artery bypass grafting, prediabetes, hypertension, gout, colon cancer, basal cell carcinoma, symptoms of muscle weakness, and loss of sensation in the hands, and significant issues with maintaining balance. His alcohol consumption was a maximum of 4 to 5 units per week, with no history of alcohol abuse.

Patient II-3 (current age 75) had medically diagnosed MSL, with cervical and upper-thoracic lipomatosis first noted around age 40. These included a large 20 cm by 20 cm lipomatous tumor growth in the midscapular region. She had been treated repeatedly over the past decade with liposuction, dermolipectomy, and laserderm with varying success, usually with recurrence. Other medical history included hypertension. She consumed no alcohol and had no history of alcohol abuse.

Patient II-4 (current age 62) has a medical history of MSL, with cervical lipomatosis first noted around age 35. She also had hypertension, a history of thyroid cancer, requiring complete thyroidectomy at the age 29, occasional muscle weakness, occasional paresthesia in her hands and feet, neural blindness in one eye, daily migraines with vomiting, and mild to moderate self-reported memory loss. She consumed no alcohol and had no history of alcohol abuse.

Patient II-5 is the proband (current age 61) and has a history of lipomatosis on her neck, forehead, shoulders, central back, and abdomen, with approximate age of onset of 35 years (see Figure 2). Other medical history included dysphagia, sleep apnea, hypertension, type 2 diabetes, vague numbness and muscle weakness in her hands, and past history of uterine teratoma, pituitary adenoma, and thyroid cancer. She consumed no alcohol.

Patient II-6 (current age 58) has a medical history of MSL with significant cervical and upper thoracic lipomatosis, since about age 40 (see Figure 2). Other medical history included hypertension, dyslipidemia, and menorrhagia requiring hysterectomy. She rarely consumed alcohol and had no history of alcohol abuse.

Patient II-7 (current age 56) had a medical history of MSL with significant cervical and upper thoracic lipomatosis, since about age 30 (see Figure 2), which were painful and restricted movement. Other medical history included dysphagia, sleep apnea, depression, anxiety, bilateral osteoarthritis of the knees, fatigue, numbness in hands and feet, occasional arm weakness, restless leg syndrome, dyskinesia, unstable gait, and significant memory loss starting in her late 40’s. She consumed no alcohol and had no history of alcohol abuse.

Patient II-8, female, was born in 1935 and died in infancy of an unspecified congenital disease.

Patient III-3 (current age 54) had lipomatosis of the neck and upper thorax, first noted at age 48. Other medical history included hypertension, pes cavus, almost daily migraine headaches occasionally with vomiting, occasional numbness and muscle weakness in hands and feet, and significant memory loss starting at age 49 and noted by her family members. She consumed no alcohol and had no history of alcohol abuse.

Patient III-5 (current age 51) had cervical lipomatosis first noted at age 45. Other medical history included migraines and occasional muscle weakness of both hands. She consumed no alcohol and had no history of alcohol abuse.

Patient III-9 (current age 42) had cervical and interscapular lipomatosis, first noted around age 25. Other medical history included an asymptomatic congenital atrial septal defect, thyroid cancer, requiring complete thyroidectomy before age 30, and constant numbness and loss of sensation in both hands. Her alcohol consumption was a maximum of 4 to 5 units per month, and she had no history of alcohol abuse.

Patient III-15 (current age 26) was reported as having early-onset glaucoma. She had no lipomatosis or neuromuscular symptoms.

### DNA Analysis

Sequencing of mtDNA identified heteroplasmy for a single variant, *MTTK* c.8344A>G, in each affected family member (see Figure 1). This variant is designated in dbSNP as rs118192098 (https://www.ncbi.nlm.nih.gov/projects/SNP/), and the reported frequency of the *MTTK* c.A8344G allele in Eurasian populations is 0.0001 (3 occurrences in 29 840 individuals). There were no other putative pathogenic variants detected in metabolic genes or in nuclear genes involved in mitochondrial function, specifically in *MFN2* encoding mitofusin 2.

## Discussion

We report one of the largest multigenerational kindreds with multiple MSL cases, each of whom carried the ultrarare *MTTK* c.8344A>G variant. Each affected family member had a clear history of adult-onset lipomatosis, of varying severity, together with inconsistent associated features. MSL has an estimated incidence of 1:25 000, with predominance in some European and Asia-Pacific populations.^1-5^ MSL typically presents in the third to sixth decades of life with the formation of soft lumps around the neck, shoulders, and upper back.^3^ Lipomatosis progressively increases in both size and distribution thereafter; growth rates are variable, often with long, interspersed periods of dormancy.^11^ The lipomatosis is not painful or tender, although compression of surrounding tissue may cause pain.^1^

Most occurrences of Madelung disease are sporadic, with no family history.^1^ Many patients have a history of alcohol overuse.^1^ Madelung disease is inconsistently associated with diabetes mellitus, hyperuricemia, hypothyroidism, liver disease, or peripheral neuropathy. The differential diagnosis of Madelung disease includes solitary benign lipoma, encapsulated lipoma, familial multiple lipomatosis, and liposarcoma. Dysphagia and dyspnea may result from laryngeal or mediastinal involvement.

A 2003 review of 272 MSL cases estimated that 28% of cases were associated with mitochondrial dysfunction, and of the few cases that were genotyped, 16% had a rare mitochondrial gene mutation.^12^ Also, while sporadic MSL is much more common in men, the high proportion of affected women in the family reported here confirms that there is no male predilection associated with the *MTTK* mutation. We note the relatively milder phenotype of the only affected male in the pedigree (subject II-2). Furthermore, the maintenance of the MSL phenotype in at least 8 maternally related relatives spanning 3 generations, in the absence of more severe neuromuscular features, supports the idea that other interacting genetic or nongenetic factors compound the tissue and organ-specific impact of the *MTTK* c.8344A>G variant.

*MTTK* codes for mitochondrial tRNA lysine (mt-tRNALys) and the c.8344A>G variant have classically been associated with myoclonic epilepsy with ragged red fibers syndrome (OMIM 545000).^13^ This variant has a frequency of 0.0001 in Eurasian populations. Among carriers of *MTTK* c.8344A>G, neuromuscular features are much more common than MSL.^11,13^ In the family reported here, there was no history of epilepsy, ataxia, or myopathy. The most consistently associated features were numbness and tingling in digits and toes and memory loss in 7/10 and 4/10 affected individuals, respectively.

Phenotypic heterogeneity between carriers of the same variant may be due to the following: (1) mitochondrial heteroplasmy; (2) a threshold effect of the mutational load; (3) interactions with other background genetic factors, including other unmeasured variants in mitochondrial or nuclear DNA; (4) gene-by-environment interactions; (5) possible epigenetic effects such as inherited changes in methylation or histone modification; or (6) unknown variables. Possible epigenetic control over the severity of the MSL presentation is suggested by altered gene expression in affected adipocytes.^14^ While beyond the scope of the present report, ex vivo studies of methylation of biopsied affected tissue samples from *MTTK* c.8344A>G carriers with MSL might be informative.

The standard of care in MSL remains surgical excision, although as noted in affected family members here, there is a high rate of relapse. Given the strong co-segregation of c.8344A>G genotype and adult-onset MSL in this family, genetic counseling could be provided to family members if requested. However, in the absence of any preventive intervention, providing a pre-symptomatic diagnosis would not appear to have any immediate clinical value, other than for patient information. If alcohol abuse is involved, abstinence or reduction of alcohol intake should be attempted, but this has not been consistently associated with regression or absence of recurrence of lipomatosis.^1^

Recent advances in mitochondrial gene therapy and mitochondrial replacement therapy with in vitro fertilization may be potential future management options. Also, coenzyme Q10 supplementation may have a benefit, given its role as a mitochondrial electron transport chain stabilizer. Clinical trials have shown no regression or stabilization of childhood-onset epilepsy or myopathy in individuals with the *MTTK* c.8344A>G variant.^15^ The effect of coenzyme Q10 is unknown in MSL.

In summary, we report a remarkable family with at least 8 individuals with *MTTK* c.8344A>G–associated MSL spanning 3 generations. A better understanding of the molecular etiologies and clinical features associated with MSL may be important in identifying specific diagnostic and management considerations, particularly in patients with mitochondrial mutations such as the *MTTK* c.8344A>G variant.

## Supplemental Material

Supplemental_File_Patient_Questionnaire – Supplemental material for Multiple Symmetric Lipomatosis (Madelung Disease) in a Large Canadian Family With the Mitochondrial MTTK c.8344A>G Variant Click here for additional data file. Supplemental material, Supplemental_File_Patient_Questionnaire for Multiple Symmetric Lipomatosis (Madelung Disease) in a Large Canadian Family With the Mitochondrial MTTK c.8344A>G Variant by Uththara Perera, Brooke A. Kennedy and Robert A. Hegele in Journal of Investigative Medicine High Impact Case Reports
