# Supplementary material for: Multiple Symmetric Lipomatosis (Madelung Disease) in a Large Canadian Family With the Mitochondrial MTTK c.8344A>G Variant
Source: J Investig Med High Impact Case Rep. 2018 Sep 29;6:2324709618802867. doi: 10.1177/2324709618802867 (PMC6166310; doi:10.1177/2324709618802867)
Supplement: Supplemental_File_Patient_Questionnaire – Supplemental material for Multiple Symmetric Lipomatosis (Madelung Disease) in a Large Canadian Family With the Mitochondrial MTTK c.8344A>G Variant [file Supplemental_File_Patient_Questionnaire.pdf]

MADELUNG – BENIGN MULTIPLE SYMMETRIC LIPOMATOSIS

QUESTIONNAIRE

MADELUNG – BENIGN MULTIPLE SYMMETRIC LIPOMATOSIS QUESTIONNAIRE

INTERVIEWER

Date (dd/mm/yyyy): ____

Interviewer Name: ____

☐ (Introduction; Reason for Phone Call; Explanation of Study; Consent)

IDENTIFICATION

Last Name (and Maiden Name)	
First Name	
Address	
Telephone Number	
E-mail Address	
Male/Female	
DOB (dd/mm/yyyy)	
Ethnicity/Background	

Name of Family Doctor	
Contact Information	

If research with your DNA reveals any medical condition relating to you, do you wish for your family doctor to be informed? ☐ Yes ☐ No

GENERAL PHYSICAL

Height (cm)	
Weight (kg)	
Waist Circumference (cm)	
Blood Pressure	

SOCIAL HISTORY

Smoking:

Have you ever smoked? If so, for how long (how many years) and how much did/do you smoke a day?

____

____

Alcohol Consumption:

Do you drink alcohol? If so, how much (per week)?

___

___

Can you describe your history of alcohol consumption in the past, particularly before/around the time of diagnosis of Madelung Disease/Lipomatosis?

___

___

MEDICAL HISTORY

Madelung Lipomatosis:

Do you have excessive weight gain or lumps around your neck, around your jawline/chin, or on the back (or anywhere else, such as your torso, waist, or lower back)?
Description (Size and Location):___

___

___

___

___

Is it symmetrical, in that it is the same when comparing the left and right side of your body? If not, please further describe the location of weight gain/lumps in relation to either side of your body.

___

___

When did they first appear?___

Have they progressively become worse/better, or changed in any way?___
Description of Changes:___

___

___

Is your skin red (erythematous) over the areas of weight gain/ lumps?

___

___

Is there any associated pain?

___

___

Have you experienced any stiffness or movement limitation in the area of weight gain/lumps?

___

___

Have you experienced any discomfort or difficulty swallowing (dysphagia)?

___

___

Any other associated symptoms or complications?

____

Can you describe your level of disability (if applicable) due to the condition?

CARDIOVASCULAR, METABOLIC AND OTHER POSSIBLE COMPLICATIONS:

Hypertension:

Do you have a history of high blood pressure? If yes, for how long?
Description:

Dyslipidemia:

Do you have a history of dyslipidemia, such as high cholesterol, high triglycerides, low HDL-C (high density lipoprotein – cholesterol), or high LDL-C (low density lipoprotein – cholesterol)?
Description:

Cardiovascular disease:

Do you have a history of heart disease, such a heart attack, chest pain (angina), stroke or transient ischemic attack (TIA), a balloon angioplasty or bypass surgery? If so, when?
Description:

Diabetes:

Have you ever been diagnosed with type 2 diabetes, non-insulin dependent diabetes, or prediabetes? If so, for how long?
Description:

Hyperuricemia:

Have you ever been diagnosed with hyperuricemia, gout, or chronic kidney disease? If so, when?
Description:____

Renal Tubular Acidosis:

Have you ever been diagnosed with renal tubular acidosis, or any other kidney disease? If so, when?
Description:____

Hepatic disease:

Have you ever been diagnosed with any liver disease, such as fatty liver or cirrhosis? If so, when?
Description:____

Hypothyroidism:

Have you ever been diagnosed with hypothyroidism? If so, when?
Description:____

Peripheral Neuropathy:

Have you ever been diagnosed with peripheral neuropathy, or had any symptoms of muscle weakness or numbness/loss of sensation? If so, when?
Description:____

Do you have any other medical conditions or health problems?
Description:____

MEDICATIONS:

Please list your current and past medications, along with the dates they were started/stopped.

SURGICAL/INTERVENTIONAL HISTORY:

Have you ever undergone any of the following procedures/interventions? If so, please give further details, including the date, the reason for intervention, the result, and any complications (if applicable).

Liposuction (Lipoplasty)

Dermolipodectomy

Laserderm

Any Other Surgeries

Physiotherapy

Have you ever had any previous genetic testing done?

FAMILY HISTORY

Do any of your family members have Madelung disease or a similar pattern of weight gain around their neck/shoulder area? If so, please specify who, and at what age they developed the condition.

Has anyone in your family had an early death, heart disease (i.e. a heart attack, angina, stroke or transient ischemic attack, balloon angioplasty or bypass surgery), or diabetes.?

Are there any other notable medical conditions that run in your family that you are aware of?

MTTK8344A.G PHENOTYPES

Do you or anyone in your family have any of the following? If yes, please describe.

Epilepsy/Seizures/Fits?

Jerky, spastic movements that you can't control?

Migraines/ Headaches? With vomiting? Difficulty concentrating?

Eye Issues/Vision Loss?

Hearing Loss?

Memory Loss?

Muscle Weakness? (In the hands/feet? Difficulty standing from sitting/squatting position?)

Issues with coordination?

Issues conducting fine movements?

Overly curved arch of feet?
